# Isotypic crystal structures of 2,6-di­bromo-*N*,*N*-bis­(4-nitro­phen­yl)aniline and 2,6-di­chloro-*N*,*N*-bis­(4-nitro­phen­yl)aniline

**DOI:** 10.1107/S1600536814010964

**Published:** 2014-07-19

**Authors:** Paul Kautny, Johannes Fröhlich, Berthold Stöger, Matthias Weil

**Affiliations:** a Institute of Applied Synthetic Chemistry, Vienna University of Technology, Getreidemarkt 9/163, A-1060 Vienna, Austria; b Institute for Chemical Technologies and Analytics, Division of Structural Chemistry, Vienna University of Technology, Getreidemarkt 9/164-SC, A-1060 Vienna, Austria

**Keywords:** crystal structure, aryl­amines, isotypism

## Abstract

In the mol­ecules of the two isotypic title compounds, C_18_H_11_Br_2_N_3_O_4_ (I) and C_18_H_11_Cl_2_N_3_O_4_ (II), the tri­phenyl­amine N atoms show no sign of pyramidalization, with marginal displacements of the N atoms from the mean plane of the three connecting C atoms: 0.0058 (13) Å for the Br compound (I) and 0.0074 (9) Å for the Cl compound (II). In the crystals, mol­ecules are linked through C—H⋯O hydrogen bonds between phenyl rings and nitro groups and by *X*⋯O (*X* = Br, Cl) inter­actions, that are shorter than the sum of the van der Waals radii, leading to a three-dimensional network.

## Abstract 2

The central ternary N atoms in the isotypic crystal structures of the substituted anilines show no sign of pyramidalization.

## Chemical context   

Aryl­amines are among the most important electron donors for functional organic materials, *e.g.* organic light emitting diodes (OLEDs) (Shirota & Kageyama, 2007 ▸; Tao *et al.*, 2011 ▸; Yook & Lee, 2012 ▸). In particular, tri­phenyl­amine-based compounds have received great attention due to their good hole-transport properties. Substituted tri­phenyl­amines are therefore highly desirable for further chemical modification, for example, cross-coupling or C—H activation.
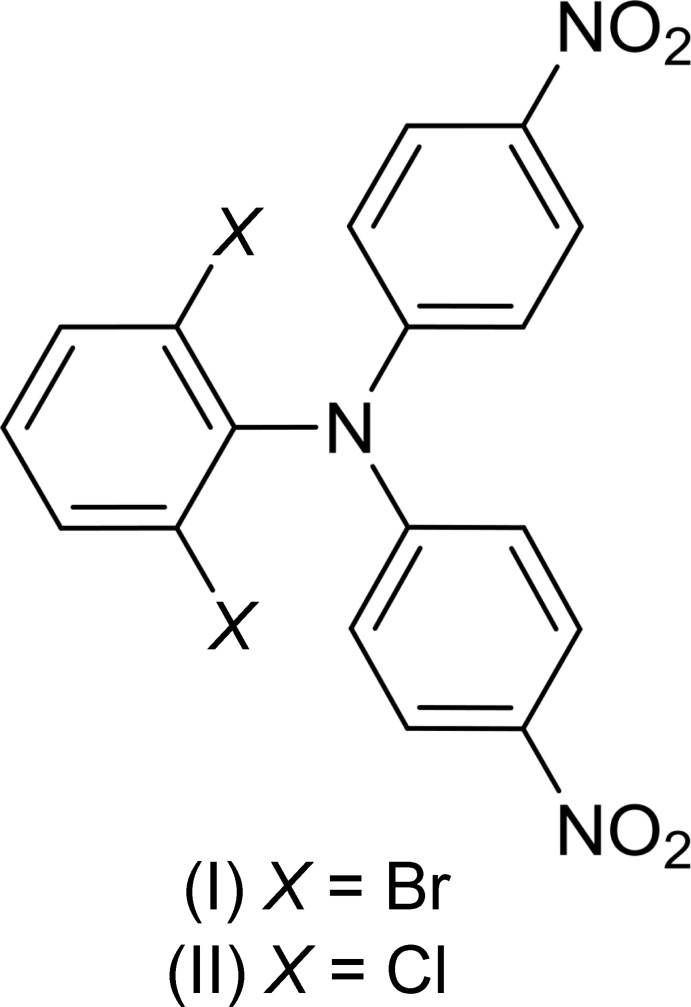


We have investigated the conversion of 2,6-dihalogenated anilines (*X* = Cl, Br) with 1-fluoro-4-nitro­benzene. Despite the sterical demand of the halogen substituents, no di­phenyl­amine inter­mediates were obtained whereas the title tetra-substituted tri­phenyl­amines (I) and (II) could be isolated and their crystal structures are reported here.

## Structural commentary   

**Figure 1:**
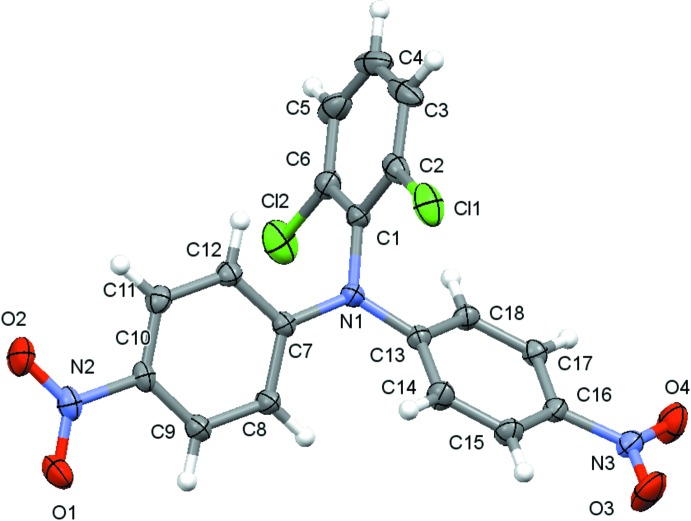
The mol­ecular structure of compound (II), with atom labelling. Displacement ellipsoids are drawn at the 70% probability level.

Representative for both structures, the mol­ecular structure of compound (II) is displayed in Fig. 1 ▸. The isotypic relation of both structures is reflected in the nearly identical bond lengths and angles in the mol­ecules of (I) and (II), and as expected, only the C—*X* distances (*X* = Br, Cl) differ significantly. The N atoms in both structures show no pyramidalization, with only marginal displacements from the planes of the bonded C atoms (C1/C7/C13) of 0.0058 (13) Å for (I) and of 0.0074 (9) Å for (II).

The dihedral angles between the benzene rings are 88.98 (7) (C1–C6)/(C13–C18), 82.07 (7) (C1–C6)/(C7–C12) and 51.97 (6)° (C7–C12)/(C13–C18) for (I). The corresponding values for (II) are 89.34 (4), 81.76 (5) and 49.41 (4)°.

The nitro groups are twisted slightly out of the plane of the benzene ring to which they are attached with dihedral angles of 8.29 (19) [(N3/O3/O4) / (C13–C18)] and 4.60 (19)° [(N2/O1/O2) / (C7–C11)] for (I). The corresponding values for (II) are 5.85 (13) and 4.81 (12)°.

## Supra­molecular features   

**Figure 2:**
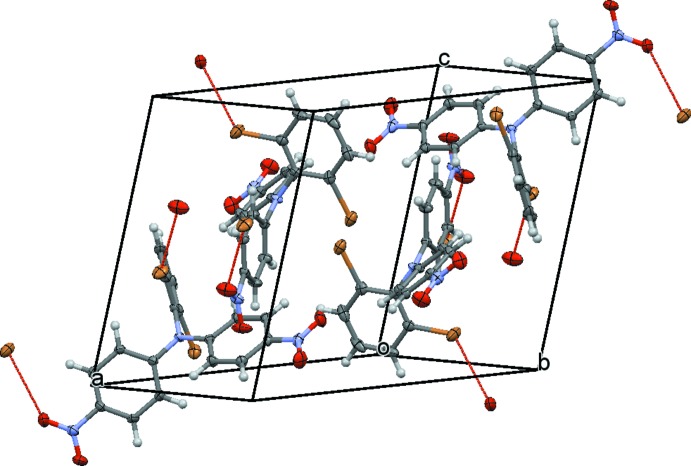
A view of the crystal packing of compound (I), sustained by Br⋯O van der Waals contacts [dashed lines; weak C—H⋯O inter­actions are also present but are not shown for clarity; colour code: O red, C grey, Br ochre, H white]. The displacement ellipsoids are drawn at the 70% probability level.

The crystal packing of the structures of both (I) and (II) is consolidated by weak —C—H⋯O—N inter­actions (Tables 1 ▸ and 2 ▸) and *X*⋯O contacts that are shorter than the sum of the van der Waals radii (Bondi, 1964 ▸) of the respective elements. For (I) the Br⋯O contact is 3.3557 (13) Å, and for (II) the Cl⋯O contact is 3.2727 (9) Å. Both types of inter­molecular inter­actions lead to the formation of a three-dimensional network (Fig. 2 ▸).

## Database survey   

A search of the Cambridge Structural Database (Version 5.35, last update February 2014; Allen, 2002 ▸) indicated the presence of 759 mol­ecules containing a tri­phenyl­amine backbone or of their metal-organic derivatives; they exclude, however, ring-closed systems such as *N*-phenyl­carbazoles or *N*-phenyl­pheno­thia­zines. None of these 759 mol­ecules possesses the substitution pattern of the title compounds, *viz*. two *para*- and one *ortho*,*ortho*-substituted benzenes with respect to the N atom. The crystal structures of one *para*-nitro-substituted tri­phenyl­amine, *viz.* tris-(4-nitro­phen­yl)amine (Welch *et al.*, 2005 ▸) and one *ortho*,*ortho*-di­chloro-substituted tri­phenyl­amine, *viz.* tris-(2,3,4,5,6-penta­chloro­phen­yl)amine (Hayes *et al.*, 1980 ▸) have been reported. As in the title compounds, in both of these mol­ecules the N atom is virtually coplanar with the three connecting C atoms. In the crystal structure of unsubstituted tri­phenyl­amine (Sobolev *et al.*, 1985 ▸), on the other hand, in three out of four mol­ecules, the N atom is located distinctly out of the plane defined by the connecting C atoms.

## Synthesis and crystallization   

Compound (I) was prepared by heating 2,6-di­chloro­aniline (405 mg, 2.50 mmol, 1.0 eq.), 1-fluoro-4-nitro­benzene (353 mg, 2.50 mmol, 1.0 eq.) and Cs_2_CO_3_ (896 mg, 2.75 mmol, 1.1 eq.) in DMSO (5 ml) at 413 K for 26 h in a capped vial using a heating block. After cooling, the reaction mixture was poured into water and the aqueous phase was extracted with CH_2_Cl_2_. The combined organic phases were dried over anhydrous Na_2_SO_4_ and concentrated under reduced pressure. Compound (I) was obtained after column chromatography (light petroleum:EtOAc 7:3) as a yellow solid (374 mg, 0.93 mmol, 74%). Yellow single crystals were grown from a CDCl_3_ solution by slow evaporation of the solvent. Spectroscopic data for compound (I) are available in the archived CIF.

Compound (II) was prepared by heating 2,6-di­bromo­aniline (627 mg, 2.50 mmol, 1.0 eq.), 1-fluoro-4-nitro­benzene (353 mg, 2.50 mmol, 1.0 eq.) and Cs_2_CO_3_ (896 mg, 2.75 mmol, 1.1 eq.) in DMSO (5 ml) at 413 K for 18 h in a capped vial using a heating block. After cooling, the reaction mixture was poured into water and the aqueous phase was extracted with CH_2_Cl_2_. The combined organic phases were dried over anhydrous Na_2_SO_4_ and concentrated under reduced pressure. Compound (II) was obtained after crystallization from an EtOH/toluene mixture as a brown solid (237 mg, 0.48 mmol, 38%). Yellow single crystals were grown from a CDCl_3_ solution by slow evaporation of the solvent. Spectroscopic data for compound (II) are available in the archived CIF.

## Refinement   

The hydrogen atoms in both structures, (I) and (II), were clearly discernible from difference Fourier maps and were refined as riding with C—H = 0.96 Å and *U*_iso_(H) = 1.2*U*_eq_(C). Experimental details are given in Table 3 ▸.

## Supplementary Material

Crystal structure: contains datablock(s) general, I, II. DOI: 10.1107/S1600536814010964/su0003sup1.cif

Structure factors: contains datablock(s) I. DOI: 10.1107/S1600536814010964/su0003Isup2.hkl

Supporting information file. DOI: 10.1107/S1600536814010964/su0003Isup4.cml

Structure factors: contains datablock(s) II. DOI: 10.1107/S1600536814010964/su0003IIsup3.hkl

Supporting information file. DOI: 10.1107/S1600536814010964/su0003IIsup5.cml

CCDC references: 1004285, 1004286

Additional supporting information:  crystallographic information; 3D view; checkCIF report

## Figures and Tables

**Table 1:** Hydrogen-bond geometry (Å, °) for (I)

*D*—H⋯*A*	*D*—H	H⋯*A*	*D*⋯*A*	*D*—H⋯*A*
C5—H1C5⋯O3^i^	0.96	2.37	3.175 (2)	141
C12—H1C12⋯O2^ii^	0.96	2.49	3.347 (2)	148

Symmetry codes: (i) 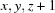; (ii) 
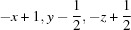
.

**Table 2:** Hydrogen-bond geometry (Å, °) for (II)

*D*—H⋯*A*	*D*—H	H⋯*A*	*D*⋯*A*	*D*—H⋯*A*
C5—H1C5⋯O3^i^	0.96	2.35	3.1950 (15)	147
C12—H1C12⋯O2^ii^	0.96	2.48	3.3304 (15)	148

Symmetry codes: (i) 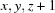; (ii) 
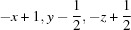
.

**Table 3:** Experimental details

	(I)	(II)
Crystal data		
Chemical formula	C_18_H_11_Br_2_N_3_O_4_	C_18_H_11_Cl_2_N_3_O_4_
*M*_r_	493.1	404.2
Crystal system, space group	Monoclinic, *P*2_1_/*c*	Monoclinic, *P*2_1_/*c*
Temperature (K)	100	100
*a*, *b*, *c* (Å)	13.4705 (7), 11.6686 (6), 11.7081 (7)	13.3117 (3), 11.5460 (3), 11.7558 (3)
β (°)	107.576 (2)	108.7971 (10)
*V* (Å^3^)	1754.39 (17)	1710.46 (7)
*Z*	4	4
Radiation type	Mo *K*α	Mo *K*α
μ (mm^−1^)	4.65	0.41
Crystal size (mm)	0.80 × 0.56 × 0.20	0.76 × 0.65 × 0.35

Data collection		
Diffractometer	Bruker *KAPPA* APEXII CCD	Bruker *KAPPA* *APEX*II CCD
Absorption correction	Multi-scan (*SADABS*; Bruker, 2013 ▸)	Multi-scan (*SADABS*; Bruker, 2013 ▸)
*T*_min_, *T*_max_	0.055, 0.390	0.74, 0.87
No. of measured, independent and observed [*I* > 3σ(*I*)] reflections	52187, 7731, 5557	29061, 4959, 4374
*R*_int_	0.045	0.031
(sin θ/λ)_max_ (Å^−1^)	0.808	0.704

Refinement		
*R*[*F* ^2^ > 3σ(*F* ^2^)], *wR*(*F* ^2^), *S*	0.034, 0.079, 1.36	0.034, 0.131, 1.39
No. of reflections	7731	4959
No. of parameters	244	244
H-atom treatment	H-atom parameters constrained	H-atom parameters constrained
Δρ_max_, Δρ_min_ (e Å^−3^)	1.00, −0.91	0.24, −0.23

Computer programs: *APEX2* and *SAINT-Plus* (Bruker, 2013 ▸), *SUPERFLIP* (Palatinus & Chapuis, 2007 ▸), *JANA2006* (Petříček *et al.*, 2014 ▸), *Mercury* (Macrae *et al.*, 2008 ▸) and *publCIF* (Westrip, 2010 ▸).

## supplementary crystallographic information

### (I) 2,6-Dibromo-N,N-bis(4-nitrophenyl)aniline Crystal data

**Table:**

C_18_H_11_Br_2_N_3_O_4_	*F*(000) = 968
*M**_r_* = 493.1	*D*_x_ = 1.866 Mg m^−^^3^
Monoclinic, *P*2_1_/*c*	Mo *K*α radiation, λ = 0.71073 Å
Hall symbol: -P 2ycb	Cell parameters from 9998 reflections
*a* = 13.4705 (7) Å	θ = 2.9–34.9°
*b* = 11.6686 (6) Å	µ = 4.65 mm^−^^1^
*c* = 11.7081 (7) Å	*T* = 100 K
β = 107.576 (2)°	Triangular prism, translucent yellow
*V* = 1754.39 (17) Å^3^	0.80 × 0.56 × 0.20 mm
*Z* = 4	

### (I) 2,6-Dibromo-N,N-bis(4-nitrophenyl)aniline Data collection

**Table:**

Bruker Kappa APEXII CCD diffractometer	7731 independent reflections
Radiation source: X-ray tube	5557 reflections with *I* > 3σ(*I*)
Graphite monochromator	*R*_int_ = 0.045
ω and φ scans	θ_max_ = 35.1°, θ_min_ = 1.6°
Absorption correction: multi-scan (*SADABS*; Bruker, 2013)	*h* = −21→21
*T*_min_ = 0.055, *T*_max_ = 0.390	*k* = −18→18
52187 measured reflections	*l* = −18→18

### (I) 2,6-Dibromo-N,N-bis(4-nitrophenyl)aniline Refinement

**Table:**

Refinement on *F*^2^	Primary atom site location: iterative
*R*[*F*^2^ > 2σ(*F*^2^)] = 0.034	Hydrogen site location: inferred from neighbouring sites
*wR*(*F*^2^) = 0.079	H-atom parameters constrained
*S* = 1.36	Weighting scheme based on measured s.u.'s *w* = 1/(σ^2^(*I*) + 0.0009*I*^2^)
7731 reflections	(Δ/σ)_max_ = 0.007
244 parameters	Δρ_max_ = 1.00 e Å^−^^3^
0 restraints	Δρ_min_ = −0.91 e Å^−^^3^
44 constraints	

### (I) 2,6-Dibromo-N,N-bis(4-nitrophenyl)aniline Special details

**Table:**

Experimental. Spectroscopic data for compound (I): ^1^H NMR (200 MHz, CDCl_3_): δ = 8.18 (d, *J* = 9.1 Hz, 4H), 7.56–7.49 (m, 2H), 7.40 (dd, *J* = 9.2, 6.6 Hz, 1H), 7.09 (d, *J* = 9.1 Hz, 4H) p.p.m.. ^13^C NMR (50 MHz, CDCl_3_): δ = 149.3 (*s*), 143.0 (*s*), 138.0 (*s*), 136.4 (*s*), 130.6 (*d*), 130.1 (*d*), 125.6 (*d*), 120.4 (*d*) p.p.m..

### (I) 2,6-Dibromo-N,N-bis(4-nitrophenyl)aniline Fractional atomic coordinates and isotropic or equivalent isotropic displacement parameters (Å^2^)

**Table:**

	*x*	*y*	*z*	*U*_iso_*/*U*_eq_	
Br1	0.573900 (13)	0.859367 (14)	0.064003 (15)	0.01908 (5)	
Br2	0.901801 (14)	1.107097 (16)	0.387459 (16)	0.02362 (6)	
O1	0.60098 (10)	1.55865 (11)	0.07605 (12)	0.0242 (4)	
O2	0.52588 (11)	1.50312 (11)	0.20583 (12)	0.0258 (4)	
O3	0.89116 (11)	0.84226 (12)	−0.27898 (11)	0.0275 (5)	
O4	1.01356 (11)	0.77627 (12)	−0.12917 (12)	0.0279 (5)	
N1	0.74647 (10)	1.04271 (11)	0.14278 (11)	0.0116 (4)	
N2	0.57864 (11)	1.48465 (12)	0.13859 (12)	0.0169 (4)	
N3	0.93586 (11)	0.83519 (12)	−0.17142 (12)	0.0166 (4)	
C1	0.74189 (12)	0.96819 (13)	0.23780 (13)	0.0119 (4)	
C2	0.67254 (13)	0.87583 (13)	0.21625 (15)	0.0146 (5)	
C3	0.67200 (13)	0.79887 (15)	0.30661 (16)	0.0199 (5)	
C4	0.73845 (14)	0.81727 (16)	0.42090 (16)	0.0225 (6)	
C5	0.80581 (14)	0.90916 (15)	0.44579 (15)	0.0191 (5)	
C6	0.80787 (13)	0.98333 (13)	0.35429 (14)	0.0150 (5)	
C7	0.70196 (12)	1.15186 (12)	0.13580 (13)	0.0108 (4)	
C8	0.72745 (13)	1.24028 (13)	0.06810 (14)	0.0143 (5)	
C9	0.68543 (13)	1.34821 (13)	0.06712 (14)	0.0150 (5)	
C10	0.61940 (12)	1.36934 (13)	0.13585 (14)	0.0132 (4)	
C11	0.59367 (12)	1.28422 (13)	0.20434 (14)	0.0132 (4)	
C12	0.63388 (12)	1.17542 (13)	0.20293 (13)	0.0128 (4)	
C13	0.79772 (12)	0.99930 (12)	0.06341 (13)	0.0114 (4)	
C14	0.75857 (13)	1.01765 (14)	−0.06029 (13)	0.0153 (5)	
C15	0.80630 (13)	0.96656 (14)	−0.13642 (14)	0.0158 (5)	
C16	0.89197 (12)	0.89691 (13)	−0.08923 (13)	0.0126 (4)	
C17	0.93330 (12)	0.87926 (13)	0.03259 (14)	0.0138 (4)	
C18	0.88567 (12)	0.93065 (13)	0.10882 (13)	0.0129 (4)	
H1c3	0.62618	0.733863	0.289983	0.0239*	
H1c4	0.737607	0.765079	0.483986	0.027*	
H1c5	0.850818	0.921597	0.525706	0.023*	
H1c8	0.774364	1.225481	0.02228	0.0171*	
H1c9	0.701637	1.408126	0.019449	0.018*	
H1c11	0.548604	1.300523	0.252057	0.0158*	
H1c12	0.615028	1.115297	0.248386	0.0154*	
H1c14	0.698685	1.065633	−0.092153	0.0183*	
H1c15	0.780239	0.979303	−0.221267	0.0189*	
H1c17	0.993928	0.83222	0.063785	0.0165*	
H1c18	0.913367	0.918984	0.19371	0.0154*	

### (I) 2,6-Dibromo-N,N-bis(4-nitrophenyl)aniline Atomic displacement parameters (Å^2^)

**Table:**

	*U*^11^	*U*^22^	*U*^33^	*U*^12^	*U*^13^	*U*^23^
Br1	0.01575 (8)	0.01677 (8)	0.02273 (9)	−0.00120 (6)	0.00282 (6)	−0.00462 (6)
Br2	0.02243 (9)	0.02083 (9)	0.02160 (9)	−0.00396 (7)	−0.00238 (7)	−0.00316 (7)
O1	0.0279 (7)	0.0125 (6)	0.0333 (7)	0.0028 (5)	0.0109 (6)	0.0051 (5)
O2	0.0296 (7)	0.0224 (6)	0.0294 (7)	0.0104 (5)	0.0153 (6)	−0.0009 (5)
O3	0.0331 (8)	0.0390 (8)	0.0116 (6)	0.0113 (6)	0.0086 (5)	0.0004 (5)
O4	0.0290 (7)	0.0353 (8)	0.0214 (6)	0.0179 (6)	0.0106 (6)	0.0037 (5)
N1	0.0163 (6)	0.0096 (5)	0.0106 (6)	0.0021 (4)	0.0063 (5)	0.0016 (4)
N2	0.0158 (6)	0.0131 (6)	0.0193 (7)	0.0025 (5)	0.0016 (5)	−0.0010 (5)
N3	0.0186 (7)	0.0181 (7)	0.0150 (6)	0.0022 (5)	0.0081 (5)	0.0015 (5)
C1	0.0141 (7)	0.0111 (6)	0.0125 (7)	0.0027 (5)	0.0069 (5)	0.0024 (5)
C2	0.0125 (7)	0.0127 (7)	0.0194 (8)	0.0026 (5)	0.0060 (6)	0.0010 (5)
C3	0.0153 (8)	0.0157 (7)	0.0320 (9)	0.0045 (6)	0.0120 (7)	0.0099 (7)
C4	0.0206 (8)	0.0251 (9)	0.0264 (9)	0.0105 (7)	0.0140 (7)	0.0151 (7)
C5	0.0201 (8)	0.0260 (9)	0.0132 (7)	0.0088 (6)	0.0077 (6)	0.0065 (6)
C6	0.0162 (7)	0.0154 (7)	0.0144 (7)	0.0017 (6)	0.0063 (6)	0.0004 (6)
C7	0.0121 (6)	0.0105 (6)	0.0099 (6)	0.0004 (5)	0.0034 (5)	0.0009 (5)
C8	0.0163 (7)	0.0132 (7)	0.0155 (7)	0.0010 (5)	0.0082 (6)	0.0020 (5)
C9	0.0167 (7)	0.0131 (7)	0.0165 (7)	0.0005 (5)	0.0068 (6)	0.0026 (5)
C10	0.0118 (7)	0.0112 (6)	0.0155 (7)	0.0017 (5)	0.0025 (5)	−0.0026 (5)
C11	0.0113 (7)	0.0146 (7)	0.0137 (7)	0.0003 (5)	0.0038 (5)	−0.0013 (5)
C12	0.0130 (7)	0.0127 (6)	0.0136 (7)	−0.0005 (5)	0.0054 (5)	0.0007 (5)
C13	0.0135 (7)	0.0101 (6)	0.0109 (6)	0.0003 (5)	0.0043 (5)	0.0002 (5)
C14	0.0162 (7)	0.0167 (7)	0.0124 (7)	0.0050 (6)	0.0036 (6)	0.0025 (5)
C15	0.0196 (8)	0.0180 (7)	0.0099 (6)	0.0037 (6)	0.0047 (6)	0.0030 (5)
C16	0.0141 (7)	0.0133 (7)	0.0123 (7)	0.0005 (5)	0.0067 (5)	−0.0007 (5)
C17	0.0140 (7)	0.0133 (7)	0.0135 (7)	0.0022 (5)	0.0033 (5)	0.0014 (5)
C18	0.0147 (7)	0.0138 (7)	0.0096 (6)	0.0010 (5)	0.0029 (5)	0.0010 (5)

### (I) 2,6-Dibromo-N,N-bis(4-nitrophenyl)aniline Geometric parameters (Å, º)

**Table:**

Br1—N1	3.0882 (13)	C5—H1c5	0.96
Br1—C2	1.8840 (15)	C7—C8	1.405 (2)
Br2—N1	3.0869 (12)	C7—C12	1.403 (3)
Br2—C6	1.8817 (16)	C8—C9	1.379 (2)
O1—N2	1.227 (2)	C8—H1c8	0.96
O2—N2	1.228 (2)	C9—C10	1.390 (3)
O3—N3	1.2237 (17)	C9—H1c9	0.96
O4—N3	1.2247 (19)	C10—C11	1.385 (2)
N1—C1	1.428 (2)	C11—C12	1.382 (2)
N1—C7	1.3995 (19)	C11—H1c11	0.96
N1—C13	1.409 (2)	C12—H1c12	0.96
N2—C10	1.457 (2)	C13—C14	1.400 (2)
N3—C16	1.462 (2)	C13—C18	1.396 (2)
C1—C2	1.398 (2)	C14—C15	1.382 (3)
C1—C6	1.396 (2)	C14—H1c14	0.96
C2—C3	1.389 (3)	C15—C16	1.383 (2)
C3—C4	1.384 (2)	C15—H1c15	0.96
C3—H1c3	0.96	C16—C17	1.381 (2)
C4—C5	1.378 (3)	C17—C18	1.383 (3)
C4—H1c4	0.96	C17—H1c17	0.96
C5—C6	1.384 (2)	C18—H1c18	0.96
			
N1—Br1—C2	52.74 (6)	N1—C7—C8	121.85 (16)
N1—Br2—C6	52.88 (5)	N1—C7—C12	119.11 (14)
Br1—N1—Br2	133.15 (5)	C8—C7—C12	118.97 (14)
Br1—N1—C1	66.74 (7)	C7—C8—C9	120.36 (17)
Br1—N1—C7	110.03 (9)	C7—C8—H1c8	119.82
Br1—N1—C13	91.63 (8)	C9—C8—H1c8	119.82
Br2—N1—C1	66.54 (7)	C8—C9—C10	119.34 (16)
Br2—N1—C7	89.49 (8)	C8—C9—H1c9	120.33
Br2—N1—C13	111.71 (8)	C10—C9—H1c9	120.33
C1—N1—C7	118.76 (14)	N2—C10—C9	119.20 (15)
C1—N1—C13	116.10 (12)	N2—C10—C11	119.18 (16)
C7—N1—C13	125.13 (13)	C9—C10—C11	121.60 (15)
O1—N2—O2	123.55 (15)	C10—C11—C12	118.96 (16)
O1—N2—C10	118.34 (16)	C10—C11—H1c11	120.52
O2—N2—C10	118.09 (14)	C12—C11—H1c11	120.52
O3—N3—O4	123.27 (16)	C7—C12—C11	120.75 (15)
O3—N3—C16	118.22 (14)	C7—C12—H1c12	119.63
O4—N3—C16	118.47 (13)	C11—C12—H1c12	119.62
N1—C1—C2	120.82 (12)	N1—C13—C14	121.39 (13)
N1—C1—C6	121.30 (13)	N1—C13—C18	118.94 (13)
C2—C1—C6	117.86 (15)	C14—C13—C18	119.55 (16)
Br1—C2—C1	119.42 (12)	C13—C14—C15	119.86 (14)
Br1—C2—C3	119.35 (12)	C13—C14—H1c14	120.07
C1—C2—C3	121.17 (14)	C15—C14—H1c14	120.07
C2—C3—C4	119.04 (16)	C14—C15—C16	119.38 (14)
C2—C3—H1c3	120.48	C14—C15—H1c15	120.31
C4—C3—H1c3	120.48	C16—C15—H1c15	120.31
C3—C4—C5	121.18 (17)	N3—C16—C15	118.75 (13)
C3—C4—H1c4	119.41	N3—C16—C17	119.29 (14)
C5—C4—H1c4	119.41	C15—C16—C17	121.85 (16)
C4—C5—C6	119.27 (14)	C16—C17—C18	118.74 (14)
C4—C5—H1c5	120.37	C16—C17—H1c17	120.63
C6—C5—H1c5	120.37	C18—C17—H1c17	120.63
Br2—C6—C1	119.26 (12)	C13—C18—C17	120.58 (14)
Br2—C6—C5	119.31 (11)	C13—C18—H1c18	119.71
C1—C6—C5	121.42 (15)	C17—C18—H1c18	119.71

### (I) 2,6-Dibromo-N,N-bis(4-nitrophenyl)aniline Hydrogen-bond geometry (Å, º)

**Table:**

*D*—H···*A*	*D*—H	H···*A*	*D*···*A*	*D*—H···*A*
C5—H1C5···O3^i^	0.96	2.37	3.175 (2)	141
C12—H1C12···O2^ii^	0.96	2.49	3.347 (2)	148

Symmetry codes: (i) *x*, *y*, *z*+1; (ii) −*x*+1, *y*−1/2, −*z*+1/2.

### (II) 2,6-Dichloro-N,N-bis(4-nitrophenyl)aniline Crystal data

**Table:**

C_18_H_11_Cl_2_N_3_O_4_	*F*(000) = 824
*M**_r_* = 404.2	*D*_x_ = 1.569 Mg m^−^^3^
Monoclinic, *P*2_1_/*c*	Mo *K*α radiation, λ = 0.71073 Å
Hall symbol: -P 2ycb	Cell parameters from 9690 reflections
*a* = 13.3117 (3) Å	θ = 3.9–30.0°
*b* = 11.5460 (3) Å	µ = 0.41 mm^−^^1^
*c* = 11.7558 (3) Å	*T* = 100 K
β = 108.7971 (10)°	Block, translucent yellow
*V* = 1710.46 (7) Å^3^	0.76 × 0.65 × 0.35 mm
*Z* = 4	

### (II) 2,6-Dichloro-N,N-bis(4-nitrophenyl)aniline Data collection

**Table:**

Bruker Kappa APEXII CCD diffractometer	4959 independent reflections
Radiation source: X-ray tube	4374 reflections with *I* > 3σ(*I*)
Graphite monochromator	*R*_int_ = 0.031
ω and φ scans	θ_max_ = 30.0°, θ_min_ = 1.6°
Absorption correction: multi-scan (*SADABS*; Bruker, 2013)	*h* = −18→18
*T*_min_ = 0.74, *T*_max_ = 0.87	*k* = −16→15
29061 measured reflections	*l* = −16→16

### (II) 2,6-Dichloro-N,N-bis(4-nitrophenyl)aniline Refinement

**Table:**

Refinement on *F*^2^	Primary atom site location: isomorphous structure methods
*R*[*F*^2^ > 2σ(*F*^2^)] = 0.034	Hydrogen site location: isomorphous structure methods
*wR*(*F*^2^) = 0.131	H-atom parameters constrained
*S* = 1.39	Weighting scheme based on measured s.u.'s *w* = 1/(σ^2^(*I*) + 0.0064*I*^2^)
4959 reflections	(Δ/σ)_max_ = 0.018
244 parameters	Δρ_max_ = 0.24 e Å^−^^3^
0 restraints	Δρ_min_ = −0.23 e Å^−^^3^
44 constraints	

### (II) 2,6-Dichloro-N,N-bis(4-nitrophenyl)aniline Special details

**Table:**

Experimental. Spectroscopic data for compound (II): ^1^H NMR (200 MHz, CDCl_3_): δ = 8.19 (d, J =9.2 Hz, 4H), 7.74 (d, J = 8.1 Hz, 2H), 7.25 (t, J = 8.1 Hz, 1H), 7.10 (d, J = 9.2 Hz, 4H) p.p.m.. ^13^C NMR (50 MHz, CDCl_3_): δ = 144.9 (*s*), 142.9 (*s*), 140.6 (*s*), 134.1 (*d*), 131.5 (*d*), 126.5 (*s*), 125.5 (*d*), 120.5 (*d*) p.p.m..

### (II) 2,6-Dichloro-N,N-bis(4-nitrophenyl)aniline Fractional atomic coordinates and isotropic or equivalent isotropic displacement parameters (Å^2^)

**Table:**

	*x*	*y*	*z*	*U*_iso_*/*U*_eq_	
Cl1	0.58310 (2)	0.86376 (2)	0.06717 (3)	0.02676 (11)	
Cl2	0.89794 (2)	1.09658 (2)	0.39011 (3)	0.02968 (11)	
O1	0.59995 (7)	1.56656 (7)	0.07725 (8)	0.0250 (3)	
O2	0.52501 (8)	1.50838 (7)	0.20576 (9)	0.0278 (3)	
O3	0.90032 (8)	0.85088 (9)	−0.27494 (8)	0.0324 (3)	
O4	1.01290 (8)	0.76623 (8)	−0.12423 (8)	0.0288 (3)	
N1	0.75055 (7)	1.04563 (7)	0.14397 (7)	0.0145 (3)	
N2	0.57820 (7)	1.49072 (7)	0.13910 (8)	0.0183 (3)	
N3	0.94009 (7)	0.83429 (8)	−0.16642 (8)	0.0174 (3)	
C1	0.74413 (8)	0.96904 (8)	0.23650 (9)	0.0145 (3)	
C2	0.67230 (9)	0.87665 (8)	0.21010 (10)	0.0185 (3)	
C3	0.66959 (9)	0.79754 (9)	0.29820 (12)	0.0260 (4)	
C4	0.73555 (11)	0.81422 (10)	0.41472 (12)	0.0297 (4)	
C5	0.80543 (10)	0.90616 (10)	0.44433 (11)	0.0253 (4)	
C6	0.80964 (9)	0.98244 (9)	0.35460 (10)	0.0184 (3)	
C7	0.70496 (8)	1.15578 (8)	0.13637 (9)	0.0133 (3)	
C8	0.73029 (9)	1.24530 (8)	0.06960 (9)	0.0166 (3)	
C9	0.68665 (9)	1.35445 (8)	0.06821 (10)	0.0172 (3)	
C10	0.61973 (8)	1.37467 (8)	0.13544 (9)	0.0151 (3)	
C11	0.59475 (8)	1.28778 (8)	0.20337 (9)	0.0158 (3)	
C12	0.63582 (8)	1.17818 (8)	0.20218 (9)	0.0151 (3)	
C13	0.80210 (8)	1.00128 (8)	0.06577 (9)	0.0137 (3)	
C14	0.76400 (9)	1.02286 (8)	−0.05822 (9)	0.0179 (3)	
C15	0.81196 (9)	0.97117 (8)	−0.13329 (9)	0.0174 (3)	
C16	0.89655 (8)	0.89650 (8)	−0.08506 (9)	0.0141 (3)	
C17	0.93636 (8)	0.87409 (8)	0.03712 (9)	0.0145 (3)	
C18	0.88927 (8)	0.92743 (8)	0.11260 (9)	0.0146 (3)	
H1c3	0.622526	0.732212	0.27836	0.0311*	
H1c4	0.732728	0.760843	0.476226	0.0357*	
H1c5	0.850452	0.917238	0.525723	0.0303*	
H1c8	0.777925	1.230913	0.024835	0.02*	
H1c9	0.702539	1.415466	0.021211	0.0206*	
H1c11	0.549454	1.3037	0.250608	0.019*	
H1c12	0.616983	1.116869	0.246644	0.0181*	
H1c14	0.704527	1.073604	−0.090899	0.0215*	
H1c15	0.786988	0.986778	−0.218011	0.0209*	
H1c17	0.995418	0.822599	0.06882	0.0174*	
H1c18	0.91652	0.913735	0.197601	0.0175*	

### (II) 2,6-Dichloro-N,N-bis(4-nitrophenyl)aniline Atomic displacement parameters (Å^2^)

**Table:**

	*U*^11^	*U*^22^	*U*^33^	*U*^12^	*U*^13^	*U*^23^
Cl1	0.01996 (17)	0.02079 (16)	0.0347 (2)	−0.00131 (9)	0.00215 (13)	−0.00737 (10)
Cl2	0.02831 (18)	0.02495 (16)	0.02653 (18)	−0.00245 (10)	−0.00402 (13)	−0.00497 (10)
O1	0.0279 (4)	0.0138 (3)	0.0335 (5)	0.0024 (3)	0.0100 (4)	0.0026 (3)
O2	0.0302 (5)	0.0245 (4)	0.0330 (5)	0.0096 (3)	0.0163 (4)	−0.0016 (3)
O3	0.0390 (5)	0.0457 (5)	0.0141 (4)	0.0168 (4)	0.0108 (4)	0.0031 (3)
O4	0.0294 (5)	0.0346 (4)	0.0233 (4)	0.0162 (4)	0.0100 (4)	0.0014 (3)
N1	0.0186 (4)	0.0124 (3)	0.0139 (4)	0.0030 (3)	0.0073 (3)	0.0024 (3)
N2	0.0166 (4)	0.0150 (4)	0.0212 (4)	0.0035 (3)	0.0031 (3)	−0.0018 (3)
N3	0.0181 (4)	0.0198 (4)	0.0160 (4)	0.0011 (3)	0.0078 (3)	0.0000 (3)
C1	0.0167 (5)	0.0128 (4)	0.0158 (5)	0.0029 (3)	0.0076 (4)	0.0025 (3)
C2	0.0161 (5)	0.0135 (4)	0.0277 (6)	0.0025 (3)	0.0093 (4)	0.0016 (4)
C3	0.0205 (5)	0.0164 (4)	0.0473 (8)	0.0057 (4)	0.0198 (5)	0.0120 (4)
C4	0.0322 (6)	0.0284 (5)	0.0390 (7)	0.0167 (5)	0.0260 (6)	0.0208 (5)
C5	0.0297 (6)	0.0309 (5)	0.0179 (5)	0.0146 (5)	0.0114 (5)	0.0087 (4)
C6	0.0205 (5)	0.0195 (4)	0.0156 (5)	0.0042 (4)	0.0064 (4)	0.0005 (4)
C7	0.0134 (4)	0.0121 (4)	0.0139 (4)	0.0009 (3)	0.0040 (3)	0.0004 (3)
C8	0.0187 (5)	0.0151 (4)	0.0192 (5)	0.0030 (3)	0.0103 (4)	0.0030 (3)
C9	0.0198 (5)	0.0135 (4)	0.0193 (5)	0.0022 (3)	0.0078 (4)	0.0027 (3)
C10	0.0139 (5)	0.0125 (4)	0.0181 (5)	0.0020 (3)	0.0039 (4)	−0.0015 (3)
C11	0.0133 (4)	0.0174 (4)	0.0176 (5)	0.0006 (3)	0.0061 (4)	−0.0010 (3)
C12	0.0145 (4)	0.0147 (4)	0.0171 (5)	−0.0002 (3)	0.0067 (4)	0.0008 (3)
C13	0.0155 (5)	0.0123 (4)	0.0133 (4)	0.0005 (3)	0.0048 (4)	−0.0001 (3)
C14	0.0200 (5)	0.0187 (4)	0.0144 (5)	0.0060 (4)	0.0045 (4)	0.0026 (3)
C15	0.0199 (5)	0.0192 (4)	0.0131 (4)	0.0035 (4)	0.0051 (4)	0.0026 (3)
C16	0.0151 (4)	0.0142 (4)	0.0144 (4)	−0.0003 (3)	0.0067 (4)	−0.0009 (3)
C17	0.0138 (4)	0.0140 (4)	0.0148 (4)	0.0013 (3)	0.0033 (4)	0.0005 (3)
C18	0.0160 (5)	0.0152 (4)	0.0114 (4)	0.0013 (3)	0.0030 (4)	0.0009 (3)

### (II) 2,6-Dichloro-N,N-bis(4-nitrophenyl)aniline Geometric parameters (Å, º)

**Table:**

Cl1—N1	2.9827 (9)	C7—C8	1.4032 (15)
Cl2—N1	2.9848 (8)	C7—C12	1.4041 (17)
O1—N2	1.2308 (13)	C8—C9	1.3855 (14)
O2—N2	1.2307 (16)	C8—H1c8	0.96
O3—N3	1.2285 (12)	C9—C10	1.3880 (18)
O4—N3	1.2219 (12)	C9—H1c9	0.96
N1—C1	1.4256 (14)	C10—C11	1.3878 (15)
N1—C7	1.3996 (12)	C11—C12	1.3803 (14)
N1—C13	1.4087 (15)	C11—H1c11	0.96
N2—C10	1.4553 (13)	C12—H1c12	0.96
N3—C16	1.4573 (15)	C13—C14	1.4027 (14)
C1—C2	1.3991 (14)	C13—C18	1.4017 (13)
C1—C6	1.3898 (13)	C14—C15	1.3805 (17)
C2—C3	1.3901 (18)	C14—H1c14	0.96
C3—C4	1.3818 (17)	C15—C16	1.3861 (14)
C3—H1c3	0.96	C15—H1c15	0.96
C4—C5	1.3801 (17)	C16—C17	1.3858 (14)
C4—H1c4	0.96	C17—C18	1.3850 (16)
C5—C6	1.3891 (17)	C17—H1c17	0.96
C5—H1c5	0.96	C18—H1c18	0.96
			
Cl1—N1—Cl2	128.74 (3)	C7—C8—C9	120.08 (11)
Cl1—N1—C1	64.63 (5)	C7—C8—H1c8	119.96
Cl1—N1—C7	110.73 (6)	C9—C8—H1c8	119.96
Cl1—N1—C13	91.15 (5)	C8—C9—C10	119.33 (10)
Cl2—N1—C1	64.21 (4)	C8—C9—H1c9	120.33
Cl2—N1—C7	91.08 (5)	C10—C9—H1c9	120.33
Cl2—N1—C13	113.45 (6)	N2—C10—C9	119.43 (9)
C1—N1—C7	118.75 (9)	N2—C10—C11	119.00 (11)
C1—N1—C13	115.72 (8)	C9—C10—C11	121.52 (9)
C7—N1—C13	125.53 (9)	C10—C11—C12	119.20 (11)
O1—N2—O2	123.46 (9)	C10—C11—H1c11	120.4
O1—N2—C10	118.27 (10)	C12—C11—H1c11	120.4
O2—N2—C10	118.25 (9)	C7—C12—C11	120.43 (10)
O3—N3—O4	122.81 (11)	C7—C12—H1c12	119.78
O3—N3—C16	118.37 (9)	C11—C12—H1c12	119.78
O4—N3—C16	118.80 (9)	N1—C13—C14	121.63 (9)
N1—C1—C2	120.56 (8)	N1—C13—C18	118.72 (9)
N1—C1—C6	121.37 (9)	C14—C13—C18	119.51 (10)
C2—C1—C6	118.06 (10)	C13—C14—C15	120.04 (9)
C1—C2—C3	121.05 (9)	C13—C14—H1c14	119.98
C2—C3—C4	119.09 (10)	C15—C14—H1c14	119.98
C2—C3—H1c3	120.45	C14—C15—C16	119.31 (9)
C4—C3—H1c3	120.45	C14—C15—H1c15	120.34
C3—C4—C5	121.24 (12)	C16—C15—H1c15	120.34
C3—C4—H1c4	119.38	N3—C16—C15	118.73 (9)
C5—C4—H1c4	119.38	N3—C16—C17	119.22 (8)
C4—C5—C6	119.01 (10)	C15—C16—C17	121.94 (11)
C4—C5—H1c5	120.49	C16—C17—C18	118.70 (9)
C6—C5—H1c5	120.49	C16—C17—H1c17	120.65
C1—C6—C5	121.48 (10)	C18—C17—H1c17	120.65
N1—C7—C8	121.84 (10)	C13—C18—C17	120.48 (9)
N1—C7—C12	118.69 (9)	C13—C18—H1c18	119.76
C8—C7—C12	119.40 (9)	C17—C18—H1c18	119.76

### (II) 2,6-Dichloro-N,N-bis(4-nitrophenyl)aniline Hydrogen-bond geometry (Å, º)

**Table:**

*D*—H···*A*	*D*—H	H···*A*	*D*···*A*	*D*—H···*A*
C5—H1C5···O3^i^	0.96	2.35	3.1950 (15)	147
C12—H1C12···O2^ii^	0.96	2.48	3.3304 (15)	148

Symmetry codes: (i) *x*, *y*, *z*+1; (ii) −*x*+1, *y*−1/2, −*z*+1/2.
